# Insecticide-Driven Patterns of Genetic Variation in the Dengue Vector *Aedes aegypti* in Martinique Island

**DOI:** 10.1371/journal.pone.0077857

**Published:** 2013-10-18

**Authors:** Sébastien Marcombe, Margot Paris, Christophe Paupy, Charline Bringuier, André Yebakima, Fabrice Chandre, Jean-Philippe David, Vincent Corbel, Laurence Despres

**Affiliations:** 1 UMR MIVEGEC (IRD 224-CNRS 5290-UM1-UM2), Institut de recherche pour le développement (IRD), Montpellier, France; 2 Laboratoire d'Ecologie Alpine (LECA), UMR 5553 CNRS- Grenoble Alpes Université, Grenoble, France; 3 Centre International de Recherches Médicales de Franceville (CIRMF), Franceville, Gabon; 4 Centre de Démoustication, Conseil Général de la Martinique, Fort de France, Martinique, France; University of Crete, Greece

## Abstract

Effective vector control is currently challenged worldwide by the evolution of resistance to all classes of chemical insecticides in mosquitoes. In Martinique, populations of the dengue vector *Aedes aegypti* have been intensively treated with temephos and deltamethrin insecticides over the last fifty years, resulting in heterogeneous levels of resistance across the island. Resistance spreading depends on standing genetic variation, selection intensity and gene flow among populations. To determine gene flow intensity, we first investigated neutral patterns of genetic variability in sixteen populations representative of the many environments found in Martinique and experiencing various levels of insecticide pressure, using 6 microsatellites. Allelic richness was lower in populations resistant to deltamethrin, and consanguinity was higher in populations resistant to temephos, consistent with a negative effect of insecticide pressure on neutral genetic diversity. The global genetic differentiation was low, suggesting high gene flow among populations, but significant structure was found, with a pattern of isolation-by-distance at the global scale. Then, we investigated adaptive patterns of divergence in six out of the 16 populations using 319 single nucleotide polymorphisms (SNPs). Five SNP outliers displaying levels of genetic differentiation out of neutral expectations were detected, including the *kdr*-V1016I mutation in the voltage-gated sodium channel gene. Association tests revealed a total of seven SNPs associated with deltamethrin resistance. Six other SNPs were associated with temephos resistance, including two non-synonymous substitutions in an alkaline phosphatase and in a sulfotransferase respectively. Altogether, both neutral and adaptive patterns of genetic variation in mosquito populations appear to be largely driven by insecticide pressure in Martinique.

## Introduction

During their life-span, mosquitoes face multiple selective pressures, including climatic constraints linked to an obligate aquatic larval stage, together with intense insecticide pressure. Insecticide pressure has led to the spreading of resistance alleles in all target species, and the managing of insecticide resistance in vectors of pathogens represents a major challenge for humanity. Originating from Africa, *Aedes aegypti* (Diptera, Culicidae) has spread throughout the tropics in historical times [1]. This mosquito species is the primary vector for dengue fever, yellow fever, and chikungunya virus in urban and sub-urban tropical areas across the world. In Martinique Island, a French overseas department located in the eastern Caribbean in the Lesser Antilles, the dengue virus (*Flaviviridae*) is transmitted by *Ae. aegypti* following an endemo-epidemic pattern and is now a major public health issue [2]. In the absence of vaccine or specific treatment against the disease, vector control by the use of chemical or biological agents against *Ae. aegypti* is currently the main method for reducing dengue transmission, in addition to environmental management and educational programs 

Over the last fifty years, larval and adult *Ae. aegypti* populations from Martinique have been intensively targeted with insecticide applications. Organochlorines such as DDT were first used since the 1950s and were replaced in the early 1990s by pyrethroid-based formulations (i.e. deltamethrin) for adult control by aerial spraying applications [3]. In addition to adult control, the organophosphate temephos (Abate®) was used for decades for larval control and there was a switch in 2009 to the bio-insecticide *Bacillus thuringiensis* *var.* *israelensis* (Bti). Because of the intense pesticide selection pressure in Martinique, vector control is now facing operational challenges with the emergence and development of insecticide resistance in *Ae. aegypti* populations [4] [5]. Resistance to organophosphates and pyrethroids has been reported since the 1980s and the 1990s respectively. A previous study has shown that deltamethrin and temephos resistances were widespread in Martinique. Molecular screening for common insecticide target-site mutations revealed the presence of the ‘‘knock-down resistance’’ V1016I *kdr* mutation at high frequency (>87%), and real time quantitative RT-qPCR showed the potential involvement of several candidate detoxification genes in insecticide resistance [3]. This study also showed that the insecticide resistance in *Ae. aegypti* populations was not homogeneously distributed across Martinique and that there were potential links existing between resistance distribution and vector control operations but also other factors such as climate and urbanization. Indeed, these factors are likely affecting the dynamics and the selection of insecticide resistance mechanisms in multiple ways [6]. The speed of resistance spreading depends on standing genetic variation in natural populations, selection intensity, local adaptation and gene flow among populations. The different climate and geological areas of Martinique with the northern part, mountainous, volcanic and rainy and the southern part slightly rugged, sunny and dry, together with the constant insecticide selection pressures and the well-developed road network that may favour passive gene flows, could lead to specific patterns of genetic diversity and population structure. The analyse of both neutral and adaptive genetic variation using genome-wide distributed molecular markers represents a powerful tool to better understand the genetic structure of these populations as well as the molecular mechanisms underlying insecticide resistance in Martinique Island.

 First, using extensive field sampling (16 populations and 31 sub-populations) in different ecotypes of the island and seven microsatellite loci, we investigated neutral patterns of genetic variability and population structure at macro and microgeographic scales across *Ae. aegypti* populations from Martinique. Then, we investigated the association between insecticide resistance (temephos and deltamethrin) and 319 single nucleotide polymorphisms (SNPs) in 6 populations, in order to identify candidate genes for temephos and deltamethrin resistances in *Ae. aegypti*. We found that both neutral (microsatellites) and adaptive (SNPs) genetic variation is affected by insecticide pressure in *Ae. aegypti* populations in Martinique.

## Materials and Methods

### Sampling localities and bioassays

**Figure 1:**
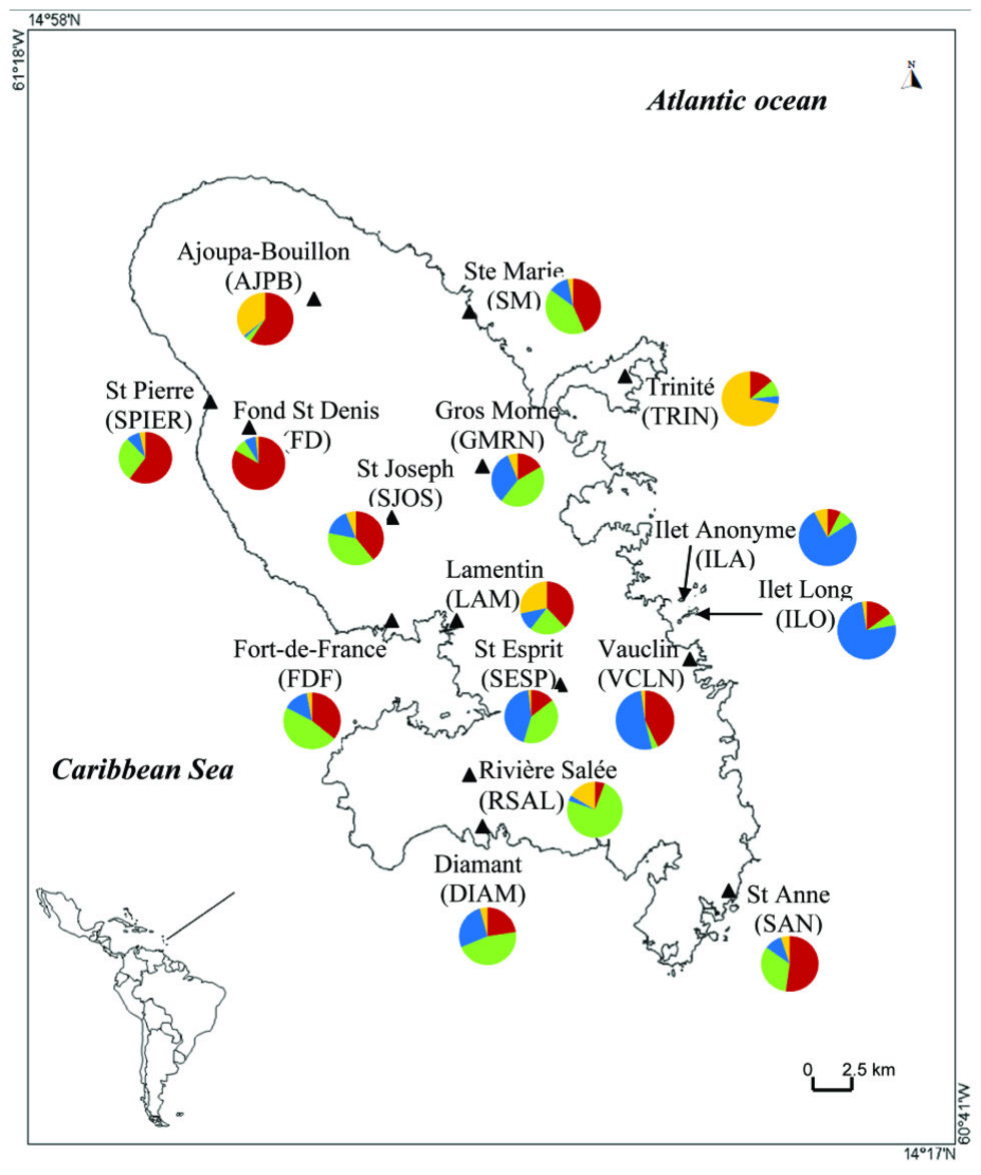
Location of *Ae. aegypti* populations sampling sites in Martinique, and the proportion of assignment to each of the four genetic clusters identified by STRUCTURE.

 Two laboratory strains from French Polynesia (Bora-Bora) and Benin (SBE) and sixteen field-caught populations were used in the study. The two laboratory strains are susceptible to all insecticides and have been used as reference strains for resistance assays. *Ae. aegypti* was sampled from individual houses as larvae or pupae in sixteen localities of Martinique from January to March 2009 (Figure 1, Table S1). Each population was constituted with about one thousands of larvae originated from 10 to 20 larval breeding sites (domestic breeding habitats) located in a 250 meters radius. Larvae and pupae were reared until the adult stage (F0 generation) and then morphologically identified. The adults were kept in a cage for copulation and females were blood-fed on rabbit (protocol CEEA-LR-13002 agreed by The French National Committee of ethics for animal experimentation) to obtain a F1 progeny. When a convenient number of F1 eggs were obtained, F0 were stored at -20°C until molecular analysis. Larvae and adults obtained from the F1 progeny were used for bioassays. The chosen populations covered most of the island ecotypes (coastal, mountainous, rural and urban) and two of them were sampled in two small islands (Ilet Long and Ilet Anonyme) located one kilometer away from the Atlantic coast (Figure 1, Table S1). This sampling also covered a large range of insecticide pressures, from no treatment up to 81 interventions between 2006 and 2009 (Table S1). No specific permits were required for mosquito collection. We confirm that most of the sampling locations were not privately-owned. We asked the owners to sample in their back yards if needed. We also confirm that the locations were not protected in any way and that the field and laboratory studies did not involve endangered or protected species.

 As described in [3], larval and adult bioassays were performed following WHO protocols [7] to measure the resistant ratios (RR_50_ and RR_95_) of twelve field-caught populations, the SBE strain (from Benin) and using the laboratory Bora-Bora strain as the susceptible reference strain. Larval and adult bioassays were carried out using temephos (Organophosphate) and deltamethrin (Pyrethroid), respectively. 

### Microsatellite genotyping and population genetic analyses

 Microsatellite genotyping and population genetic analyses were implemented at the macro-geographic level using 16 F0 samples of 30 females and 10 males. In 3 localities, Fort-de France, St-Anne and Vauclin, individuals from 10, 4 and 4 distinct larval breeding sites respectively, were used for a micro-geographic level analysis. DNA was extracted from adult mosquitoes of both sexes using 2% CTAB as described in [8]. DNA pellets were resuspended in 20 µL sterile water and stored at -20 °C until analysis. Individual genotypes were scored at a set of seven microsatellite markers 34/72, AEDGA, AED19, AEDC, A10, 38/38 and H08. The microsatellites and PCR conditions have previously been detailed [9,10]. DNA was amplified in a GeneAmp 9600 thermal cycler (PerkinElmer, USA). PCR products were analyzed using ABI Prism™ 3100 (Applied Biosystems), and genotyped with GeneMapper (Applied Biosystems) (for details see 9). The microsatellite 38/38 was not polymorphic in our populations and was not kept for further analyses.

Genetic diversity by locus and sample and overall was characterized by estimating the number of alleles and allelic richness (Rs, [11]) using the software FSTAT2.9.3.2 [12]. Linkage disequilibrium, deviations from Hardy-Weinberg equilibrium (HWE) and genetic differentiation indices were assessed using GENEPOP4.7 software [13]. *F*_*IS*_ and *F*_*ST*_ estimates were calculated according to [14] and tested for statistical significance with exact tests available in GENEPOP4.7. The overall significance of multiple tests was estimated by Fisher’s combined probability tests. Nominal significance levels for multiple testing were corrected using the sequential Bonferroni procedure [15]. Genetic isolation by geographic distance was assessed with Mantel’s test in GENEPOP4.7, using the correlation between genetic and geographic distances by the regression of pairwise *F*_*ST*_ /(1- F_*ST*_) on the natural logarithm (Ln) of straight-line geographic distance [16]. Genetic structure was also studied at the macro and micro-geographic levels by examining the partition of neutral genetic variation using an AMOVA (Analysis of Molecular Variance, Arlequin 3.1 [17]). A bayesian clustering method implemented in STRUCTURE 2.3 [18] was used to assign individuals from all 16 populations to a pre-determined number of clusters (K=1-16) based on multi-locus microsatellite data. For each run, a burn-in period of 10,000 steps was followed by 30,000 iterations under the admixture model and the assumption of correlated allele frequencies among populations. To assist the clustering, sampling locations were used as prior information (LOCPRIOR model). For each K of 1–16, 20 runs were performed. Estimated log probabilities (Ln P(D)) were averaged across runs and compared to determine the posterior probability of each K. K with the highest likelihood was retained. Pearson’s correlation between genetic diversity estimates (allelic richness and *F*_IS_) and environmental variables (altitude, human density) and resistance ratio were tested using the “stat” package in the R software version 2.10 [19]. 

### SNP genotyping

Six out of the 16 populations (GRMN, ILA, ILO, LAM, RSAL and SAN) representative of the range of insecticide resistance levels observed in Martinique were analysed for 319 SNPs. 318 of these SNPs were developed in the *Ae. aegypti* laboratory strain Bora-Bora using transcriptomic data in [20]. Briefly, short cDNA tags were obtained from pools of larvae and sequenced using a Genome Analyzer I (Illumina Corporation, USA). The reads were filtered, mapped to *Ae. aegypti* genome assembly AaegL 1.1 (see VectorBase, http://aaegypti.vectorbase.org/) and 1520 SNPs with a coverage > 10x and a minimal allelic frequency of 5% were detected. The 318 SNPs used in this study were successfully transferred and genotyped in the natural *Ae. aegypti* populations of the Martinique Island [20]. They were distributed in 226 out of the 4,758 supercontigs in the reference genome. The knock down *kdr*-V1016I mutation in the voltage-gated sodium channel gene was also included in the SNP panel, as it was the only one found in a previous scan in Martinique populations among the four I1011M, I1011V, V1016I and V1016G *kdr* mutations [3] [21] . DNA was extracted from whole larvae or adults (30-40 individuals per population) from the F1 generation (also used for bioassays), using the DNeasy tissue Kit (Qiagen) according to the manufacturer’s instructions. DNA concentration was determined for each sample with a NanoDrop^TM^ ND-1000 (NanoDrop Technologies), normalized to 50ng/µl and genotyped using the DNA Illumina GoldenGate® array genotyping platform at the Genotyping Center GENTYANE in Clermont-Ferrand, France(http://www.ibisa.net/plateformes/GENTYANE,101.html). The fluorescent signal intensities of the specific alleles were determined by the BeadExpress Reader (Illumina Inc), and quantified and matched to genotype classes using GenomeStudio v3.1.14 (Illumina Inc) with manual adjustment when necessary. To ensure a good reliability of the genotyping data we used the GenCall50 (GC50) which indicates the reliability of each genotype call, the GenTrain which measures the cluster quality of the SNPs, and the call rate (CR) which represents the fraction of the samples that has been successfully genotyped for a given SNP. We kept SNPs with GC50 higher than 0.35, GenTrain higher than 0.50 and CR higher than 0.50.

### Detection of signature of positive selection (Fst outliers)

To detect SNPs displaying higher than expected differentiation under neutrality between the 6 populations (outliers) we used BayeScan v3 (freely available at http://cmpg.unibe.ch/software/bayescan/). This Bayesian method is based on a logistic regression model in which each logit value of genetic differentiation Fst*ij* at a given locus *i* in population *j* is decomposed as a linear combination of α*i* and β*j*, corresponding to a locus effect and to a population effect, respectively. To test for the signiﬁcance of α*i* for each locus, the posterior probability that α*i* is different from zero is estimated using a Reversible Jump MCMC algorithm [22]. Priors for α*i* and β*j* were Gaussian distributions with means 0 and -2 respectively and standard errors 1 and 1.8 as in [23]. The estimation of model parameters was automatically tuned on the basis of short pilot runs (10 pilot runs, length 2000). Preliminary tests indicated that a burn-in of 10,000 iterations was enough to attain convergence. We used a sample size of 10,000 and a thinning interval of 50, as suggested by [22], resulting in a total length of the chain of 500,000 iterations. Four independent runs were performed to check the consistency between detected outliers. The loci were ranked according to their estimated posterior probability value and all loci with a Bayes factor >3 were retained as outliers.

### Association with insecticide resistance

RR_95_ and RR_50_ were highly correlated (r^2^ = 0.92 and 0.90, both *P* < 0.0001, for temephos and deltamethrin respectively) and we used RR_95_ in association analysis in order to maximize the range of RR variation between the populations. Association analyses between RR_95_ and SNPs were performed using the least squares fixed effects linear model implemented in the software TASSEL v3 [24]. This model tests for association between genotypes at each segregating marker and phenotype values, while accounting for population structure by using the 3 first principal components of a PCA derived from genetic markers as covariates. *P* values were corrected for multiple comparisons by permutation tests with 1000 permutations and 0.05 confidence level.

### Markers location in the reference genome and candidate genes identification

All SNPs detected either as *Fst* outliers, or as being significantly associated with a resistance phenotype, were blasted on the reference genome of *Ae. aegypti* (http://aaegypti.vectorbase.org/) to locate them inside or at proximity of potential candidate genes. The length of linkage disequilibrium can strongly vary from a genomic region to another and from a population to another, so that it is very difficult to assess an overall disequilibrium length in natural populations. In a closed population (a laboratory *Ae. aegypti* strain) under a standard pure-drift demographic model, overall linkage disequilibrium was estimated to be 20 Kb for neutral markers [25]. In a genomic region under selection, the amount of linkage disequilibrium can be considerably greater than that predicted for a neutral region. Furthermore, in natural population, gene flow can produce significant levels of disequilibrium. Therefore we have arbitrarily chosen a conservative threshold of 500 Kb to search for candidate genes potentially under selection in the vicinity of outlier markers, and all the genes present up to 500 Kb each direction from the detected SNP were considered as potentially involved in the resistance phenotype and discussed.

## Results

### Bioassays

Bioassays showed that the twelve tested populations from Martinique were resistant to temephos and deltamethrin in comparison with the laboratory susceptible Bora-Bora strain and the SBE strain from Benin (Table 1). However, the resistance observed against temephos and deltamethrin was heterogeneous in the island. For temephos, RR_50_ ranged from 13 fold (SJOS) to 36-fold (GMRN) and RR_95_ ranged from 26-fold (SJOS) to 153-fold (VCLN). The populations from the two islets showed a lower resistance level to temephos with RR_50_ of 5-fold for both islets, and RR_95_ of 17 and 21-fold for ILA and ILO, respectively. Mortality after 24 h deltamethrin exposure ranged from 19% for AJPB to 90% for the RSAL population compared to the 100% mortality in the susceptible strains Bora-Bora and SBE. For each insecticide, resistance ratios were not significantly explained by the number of insecticide treatments, or by other environmental variables such as the type of locality (rural, urban), human density or altitude (Table S1). 

**Table 1:** Resistance status of *Aedes aegypti* populations of Martinique to temephos (larvae) and deltamethrin (adults).

Insecticide	**Temephos**			**Deltamethrin**			
Strain	RR_50_ (ci RR_50_)	RR_95_ (ci RR_95_)	n	RR_50_ (ci RR_50_)	RR_95_ (ci RR_95_)	Mortality (24h)	n
Bora^1^	¤	¤	995	¤	¤	100%	204
SBE	0.7 (0.5-0.8)	0.83 (0.8-0.9)	502	1 (0.93-1)	0.9 (0.9-1)	100%	101
SPIER	**13 **(12-15)	**54 **(42-75)	498	**4 **(3.9-4.2)	**5.8 **(5.3-6.7)	70%	97
RSAL	**29 **(26-33)	**82 **(65-110)	501	**3.7 **(3.6-3.8)	**4.7 **(4.3-4.8	90%	99
VCLN	**27 **(25-32)	**153 **(117-211)	699	**5.7 **(5.1-6.8)	**7.1 **(6 -10.5)	20%	99
DIAM	**23 **(21-26)	**90 **(73-117)	702	**5.4 **(5-6.1)	**6.2 **(5.4-8.5)	34%	99
LAM	**32 **(28-36)	**139 **(113-176)	701	**5.6 **(6.8)	**8 **(6.5-12.8)	55%	94
GRMN	**35 **(30-45)	**149 **(102-217)	697	**5.3 **(4.9-6.1)	**6.9 **(5.9-9.6)	64%	101
SAN	**19 **(18-22)	**56 **(45-73)	699	**6.7 **(5.7-8.6)	**12.2 **(9-21.5)	42%	98
SJOS	**13 **(11-16)	**27 **(16-42)	696	**5.8 **(5.2-7)	**8.5 **(6.8-13.4)	29%	95
FDF	**15 **(14-16)	**36 **(30-45)	702	**6.4 **(5.5-8.6)	**9.4 **(7-18.2)	23%	101
AJPB	**15 **(13-17)	**54 **(43-74)	600	**5.9 **(5.2-6.9)	**10.3 **(8.2-15.8)	19%	103
Bora2^1^	¤	¤	602	¤	¤	100%	100
ILA	**5 **(3-7)	**17 **(7-39)	900	**6.7 **(6.6-6.8)	**7.9 **(-7.5-9)	27%	100
ILO	**5 **(4-5)	**21 **(17-29)	900	**5.1 **(4.9-5.4)	**7.9 **(7.8-8.4)	38%	101

1 Bioassays were performed in two series corresponding to two generations of the laboratory susceptible Bora-Bora strain (Bora and Bora2). RR: resistant ratio; ci: confidence interval; LC_50_ and LC_95_ in mg/L were 0.003 and 0.06 respectively for Bora and 0.005 and 0.01mg/L for Bora2 with temephos. KD_50_ and KD_95_ were 14 and 21 min respectively (11 and 18min for Bora2) with deltamethrin.

### Microsatellite polymorphism analysis

#### Genetic variability and Hardy-Weinberg expectations

A total of 31 populations were successfully genotyped with the 7 microsatellites markers that were all polymorphic except 38/38 that was not kept for further analyses. Genetic diversity was relatively homogeneous among the different populations tested at both macro and micro-geographic levels (Table 2 and Table S2). Average allelic diversity was low (2.33 alleles/locus, range 1.73-2.38) as compared to the diversity found in African populations [10], but comparable to the diversity found in south American populations [26]. At the macro-geographic scale, when the populations were considered together, important heterozygote deficit were detected (34/72 and H08, and all loci; Table 2), suggesting that Martinique is not a single panmictic unit. However, within localities, departures from Hardy-Weinberg expectations and significant heterozygote deficits were detected only 3 times: in GRMN at locus 34/72, and in AJB and RS when all loci were considered together (Table 2). Overall, these results indicate that each sampled site can be considered as a panmictic population. At the micro-geographic level and considering the microsatellite markers individually, significant deviations were detected only in Fort-de-France samples (Table S2). Genetic diversity indices (*Nall*, *Rs* and *F*_*IS*_) were not correlated (all *P* > 0.05) with the environmental variables measured (altitude, human density). However, allelic richness was negatively correlated with the level of resistance to deltamethrin (t = -2.66, df = 10, *P* = 0.02, r = -0.64) underlying a significant decrease of diversity in the most resistant populations. Furthermore, *F*_*IS*_ was significantly correlated with the level of resistance to temephos (t = 2.43, df = 10, *P* = 0.035, r = 0.609) showing an increased consanguinity in populations more resistant to temephos. 

**Table 2:** Genetic variability and goodness of fit to Hardy-Weinberg expectation in *Ae. aegypti* populations at the macro-geographic level in Martinique.

		1 n=30	2n=30	3 n=30	4 n=30	5n=30	6 n=30	7 n=30	13 n=30	18 n=30	19 n=30	20 n=30	21 n=30	22 n=30	26 n=30	30 n=30	31 n=30	all
**3472**	*N_all_*	2	4	2	3	5	3	3	2	3	3	3	3	2	2	2	2	5
	*R_s_*	1.33	3.29	1.36	2.96	3.26	2.36	2.76	2.00	2.72	2.71	2.78	2.71	1.78	2.00	2.00	2.00	2.66
	*F_IS_*	0.000	0.285	0.000	0.233	0.264	**0.774**	-0.147	-0.364	0.137	-0.114	0.099	-0.130	-0.042	-0.515	-0.862	-0.262	**-0.009**
**Aedga**	*N_all_*	1	1	1	1	1	1	1	1	1	1	1	2	1	1	1	1	4
	*R_s_*	1.00	1.00	1.00	1.00	1.00	1.00	1.00	1.00	1.00	1.00	1.00	1.61	1.00	1.00	1.00	1.00	1.07
	*F_IS_*	NA	NA	NA	NA	NA	NA	NA	NA	NA	NA	NA	-0.020	NA	NA	NA	NA	0.328
**A10**	*N_all_*	3	4	3	3	2	4	4	4	3	3	2	4	3	3	4	4	6
	*R_s_*	3.00	3.55	3.00	2.56	2.00	3.62	2.89	2.67	2.56	2.56	2.00	3.15	2.71	2.81	3.62	3.10	3.23
	*F_IS_*	-0.397	0.004	-0.103	-0.324	-0.063	0.138	0.092	0.077	-0.202	-0.217	0.392	0.020	-0.037	0.101	0.288	-0.021	-0.035
**Aed19**	*N_all_*	2	2	2	2	2	2	2	2	2	2	2	2	2	2	2	2	2
	*R_s_*	2.00	2.00	2.00	2.00	2.00	2.00	2.00	2.00	2.00	2.00	2.00	2.00	2.00	2.00	2.00	2.00	2.00
	*F_IS_*	0.366	0.116	-0.166	-0.241	0.102	-0.102	-0.080	-0.244	0.067	-0.120	-0.017	0.191	0.198	0.231	-0.200	0.087	-0.010
**Aedc**	*N_all_*	3	2	1	2	2	2	2	2	2	3	3	3	2	4	4	1	6
	*R_s_*	2.59	1.89	1.00	1.93	1.99	1.93	1.97	1.38	1.97	2.04	2.51	1.89	1.76	3.06	2.03	1.00	2.16
	*F_IS_*	-0.118	-0.077	NA	-0.098	-0.191	-0.098	-0.137	0.000	0.151	-0.040	0.380	0.663	-0.040	-0.008	0.325	NA	-0.012
**H08**	*N_all_*	3	3	2	2	3	3	3	3	3	2	3	3	3	2	3	3	4
	*R_s_*	2.45	2.57	2.00	2.00	2.36	3.00	2.92	2.63	2.98	2.00	3.00	2.73	2.82	2.00	2.84	2.37	2.85
	*F_IS_*	0.649	0.004	-0.125	-0.250	-0.288	-0.251	-0.039	-0.192	0.345	-0.012	0.197	0.345	0.217	0.386	0.146	-0.157	**0.001**
**Mean across all loci**	*N_all_*	2.33	2.67	1.83	2.17	2.50	2.50	2.50	2.33	2.33	2.33	2.33	2.83	2.17	2.33	2.67	2.17	4.50
	*R_s_*	2.06	2.38	1.73	2.08	2.10	2.32	2.26	1.95	2.21	2.05	2.22	2.35	2.01	2.15	2.25	1.91	2.33
	*F_IS_*	**0.004**	0.069	-0.124	-0.134	-0.040	0.098	-0.041	-0.169	0.106	-0.111	**0.190**	0.152	0.087	0.029	-0.102	-0.088	**-0.014**

1 Ajoupa-Bouillon; 2: Saint-Pierre; 3: Fond-Saint-Denis; 4: Sainte-Marie; 5: Trinité; 6: Gros-Morne; 7: Saint-Joseph; 13: Fort-de-France; 18: Lamentin; 19: Saint-Esprit; 20: Rivière Salée; 21: Diamant; 22: Sainte-Anne 1; 26: Vauclin 1; 30: Ilet Anonyme; 31: Ilet Long. n: sample size. *N*_*all*_: number of scored alleles; *R*_*s*_: Allele richness; *F*_*IS*_: Inbreeding coefficient; In bold: significant deficit in heterozygotes (*P* < 0.05) after Bonferroni correction.

### Genetic differentiation and population structure

The global genetic differentiation index *F*_*ST*_ was low (0.079) but the populations were significantly structured both at the macro-geographic and at the micro-geographic scales (Table 3). Pairwise *F*_*ST*_ values were significant for 69% of the population pairs tested. Genetic and geographic distances were correlated at the Martinique scale (Mantel test; *P* = 0.009) and at the local scale in Vauclin (*P* = 0.042), but not in Fort-de-France and Sainte-Anne (*P* > 0.5). Four genetic clusters were identified in Martinique (Figure 1), one being mostly represented in the two islets and their closest eastern Vauclin population on the main island, and another corresponding to populations around the gulf of Fort-de-France (urban area). All the populations in Martinique were highly admixed, suggesting important gene flow across populations. The most homogenous populations were the two islets assigned >80% to one cluster and Fond-St-Denis assigned >90% to another cluster.

**Table 3:** Genetic differentiation estimated with 6 microsatellite markers at two geographic levels.

	**Number of populations**	***F_ST_^a^***	***P***	**% pairwise differentiation^b^**
Macro-geographic level	16	0.0719	**< 10^-4^**	69
Micro-geographic level				
Fort de France	10	0.0839	**< 10^-4^**	69
Sainte-Anne	4	0.0741	**< 10^-4^**	100
Vauclin	4	0.0532	**< 10^-4^**	83

a Fst indices estimated by population groups and *P*, probability associated with Fisher’s exact test

b Average pairwise population percentages showing a significant genetic differentiation after Bonferroni correction using a threshold of *P*<0.05

### SNP diversity

Average allelic diversity per locus per population was 1.76 (range 1.6-1.9) and the 6 populations were at the HW equilibrium except for a slight deficit in heterozygotes in Rivière Salée. The global *Fst* = 0.031 was low, suggesting large gene flow among these populations; the structure analysis based on SNPs identified the same 4 main clusters as identified based on microsatellites (data not shown). Pairwise *Fst* were generally smaller for SNPs than for microsatellites (Table S3).

### Detection of Fst outliers and association tests

Five out of the 319 SNPs were identified as *Fst* outliers across the 6 populations tested in Martinique (Table 4), including the *kdr*-V1016I mutation in the voltage-gated sodium channel. However, the *kdr* mutation was not detected as significantly associated with deltamethrin resistance. The resistance allele is found at high frequency in all populations on the main island (85% to 92%), confirming previous report [3], which may explain the failure of the GLM regression to detect significant association between deltamethrin resistance and the frequency of the resistant allele. From the five *Fst* outliers, two were significantly associated with deltamethrin resistance, and one with temephos resistance. Five other markers were associated with deltamethrin resistance and four other were associated with temephos resistance (Table 4). Taken separately, each marker explained from 9 to 17% of the resistance variability observed between populations.

**Table 4:** Summary of the SNPs detected as outliers in BayeScan analysis and/or as associated with a resistance phenotype.

**SNP name (supercontig_gene)**	**Bayescan outlier**		**Associated resistance phenotype**	**perm_p**	**R^2^**	**Supercontig number**	**gene**	**fonction**	**5'-3' position**	**alleles**	**aa change**	**Supercontig size**
1.4_AAEL000245	yes		deltamethrin	0.006	0.093	1.4	AAEL000245	conserved hypothetical protein	5094449	[T/C]		5177111
1.250_AAEL007363	yes		deltamethrin	0.001	0.143	1.250	AAEL007363 _(3' UTR)_	leucine-rich transmembrane protein	337073	[A/C]		1628417
1.636_NA0299	yes		temephos	0.001	0.170	1.636			381176	[T/C]		653825
1.200_NA0113	yes					1.200			1695291	[T/G]		1852562
1.186_*kdr*-V1016I	yes					1.186	AAEL006019	voltage-gated sodium channel	115680	[A/G]	Ile-Val	1957664
1.1168_AAEL014562_A			deltamethrin	0.001	0.135	1.1168	AAEL014562	60S ribosomal protein L12	182837	[T/C]		191961
1.1168_AAEL014562_B			deltamethrin	0.006	0.092	1.1168	AAEL014562	60S ribosomal protein L12	183099	[A/G]		191961
1.541_AAEL011089			deltamethrin	0.003	0.105	1.541	AAEL011089	Ribonucleoprotein	250291	[G/C]		819817
1.68_AAEL002794			deltamethrin	0.024	0.080	1.68	AAEL002794 _(3' UTR)_	hypothetical protein	1416497	[A/C]		2950385
1.70_AAEL002875			deltamethrin	0.029	0.101	1.70	AAEL002875	hypothetical protein	79260	[T/G]		2929944
1.83_AAEL003298			temephos	0.016	0.115	1.83	AAEL003298	alkaline phosphatase	254086	[T/A]	Thr-->Ser	2974912
1.414_AAEL009633			temephos	0.001	0.154	1.414	AAEL009633	conserved hypothetical protein	1016060	[T/C]		1096716
1.355_AAEL008898			temephos	0.03	0.092	1.355	AAEL008898	sulfotransferase	665884	[A/C]	Asp-->Lys	1256691
1.1002_AAEL014080			temephos	0.002	0.131	1.1002	AAEL014080	Aldehyde deshydrogenase	176807	[A/G]		296325

a Fst indices estimated by population groups and *P*, probability associated with Fisher’s exact test

b Average pairwise population percentages showing a significant genetic differentiation after Bonferroni correction using a threshold of *P*<0.05

### Markers location in the reference genome and candidate genes identification

Most of the SNPs identified were either inside a gene annotated in Vectorbase, or in the flanking 3’ extremity of a gene, which is not surprising because SNPs have been designed based on transcriptome sequences anchored on the 3’ end of transcripts [27]. The seven SNPs associated with deltamethrin resistance corresponded to six genes encoding respectively a leucin-rich membrane protein, two proteins associated with ribosomes, and three hypothetical proteins of unknown function. All SNPs were synonymous. Among the five SNPs associated with temephos resistance, two were non-synonymous mutations in an alkaline phosphatase and in a sulfotransferase, two were synonymous mutations in an aldehyde dehydrogenase and an unknown protein, and the last one was in a non-coding region. Genes located on the same supercontig and within ± 500 Kb from a marker associated with resistance are presented in Table S4. Among these, genes encoding enzymes potentially involved in detoxification processes such as transferases, phosphatases and dehydrogenases were identified. Genes encoding transcription factors and miRNA potentially regulating gene expression were also identified.

## Discussion

### Population structure at local and global scales

Our study showed a low genetic diversity in *Ae. aegypti* populations from Martinique compared with levels observed in other parts of the world, especially in native African populations [9,10], but was consistent with a previous study based on isoenzymes in this Island [28]. The loss of genetic diversity observed here can be explained by the founder effect that occurred during the introduction of the mosquito in the Caribbean. Furthermore, this low genetic diversity may have been maintained since its introduction in the 17th century by several factors such as recurrent demographic crashes linked to the specific environmental variations in Martinique (i.e. rainfall and droughts) and more recently by anthropogenic actions such as vector control. Because of the recurrent Dengue epidemics occurring in Martinique, vector control (i.e. source reduction and insecticide applications) is intense and may induce local bottleneck effects and a reduction of the genetic diversity in *Ae. aegypti* populations. In support to this hypothesis, we found significant correlations between neutral genetic diversity estimates and insecticide resistance ratio: allelic richness was negatively correlated to deltamethrin resistance, suggesting that population reduction following insecticide treatments leads to allelic loss. Furthermore, *F*_*IS*_ was positively correlated with temephos resistance, meaning that more resistant populations are more consanguineous, a result expected if only individuals carrying resistance alleles and their progeny can survive. 

Despite an overall low genetic differentiation among sites, most pairwise *Fst* were significant and there was a significant pattern of isolation-by-distance (IBD) at the whole Martinique scale. This suggests that gene flow mostly occurs at a local scale with high admixture occurring among neighbour sites. Furthermore, four genetic clusters were identified, with marked differences in the proportion of assignation of individuals to each cluster from a site to another. Northern populations were mostly assigned to one cluster (red on Figure 1) and Southern populations were more admixed with a predominance of another cluster (green on Figure 1). The genetic drift observed in Fond-Saint-Denis (mostly red cluster) and in the Islets (mostly blue cluster) could be explained by their geographic isolation, with restricted access in the mountainous area and only accessible by boat, respectively. At a more local scale, the IBD pattern was marginally significant in only one out of three sites analysed (Vauclin, *P* = 0.042), supporting the effect of genetic drift acting on mosquito populations. Genetic drift at local scale could be due to the recurrent insecticide applications leading to local bottlenecks. Furthermore, flight activity of *Ae. aegypti* is limited to few hundred meters [29] which is likely to increase the neutral genetic differentiation between the populations through genetic drift. No significant correlation was found between diversity estimates and environmental variables such as altitude or human density. 

### Outlier detection and association with insecticide resistance

The genome scan analysis was performed on 6 populations representative of the diverse mosquito habitats found across Martinique Island, and of the most extreme insecticide resistance ratio values observed. BayeScan detected five outliers out of 319 SNPs, all with positive α*i* values, indicative of directional selection. These included the knockdown resistance *kdr* mutation (V1016I), which is a well-characterized mechanism of resistance to pyrethroid insecticides in insects (including *Ae. aegypti*) and is caused by a point mutation of the *para*-type sodium channel [30], and three other genomic regions that were associated with resistance to either temephos or deltamethrin. Therefore, only one out of five BayeScan outliers was not related to insecticide pressure. This outlier was not found significantly associated to altitude or to human density, but it might be linked to other environmental or ecological pressures that were not measured in the present study, including climate and/or agricultural practices. Despite a highly diversified landscape in Martinique, and a complex life cycle involving an aquatic larval stage and an aerial adult stage, the mosquito genome appears to be more constrained by insecticide pressure than by any other environmental pressure. Although identified as an outlier by the BayeScan approach, the *kdr* mutation V1016I was not significantly associated with deltamethrin resistance; this could be due to the fact that this mutation was present at high frequency in all the main island populations (>85%, as also found in [3]), decreasing the power of the association study. Furthermore, other *kdr* mutations such as the *kdr* mutation F1534C found at a relative high frequency in Grand Cayman Island and shown to be linked with DDT and permethrin resistance [31] might also be involved in pyrethroid resistance in Martinique, and their occurrence should be searched for in Martinique. Association study revealed that nine additional SNPs not detected as outliers by BayeScan were significantly correlated with resistance to either insecticide. BayeScan is a conservative approach and is sensitive to the level of genetic differentiation across populations: if genetic differentiation is too small, the Reversible Jump MCMC algorithm used to estimate posterior probabilities can fail to reach convergence, and if it is too high, the power to detect outlier loci is low. In the association study, population structure is taken into account as a fixed effect in the general linear model fitting phenotypic to genotypic value at each locus under investigation. As it is a correlative approach, it is not sensitive to population processes such as drift, migration, and demographic fluctuations. On another hand, this correlative approach might detect false positives. We independently detected two markers significantly associated with deltamethrin resistance located at two different positions in the same gene (AAEL014562) suggesting that this association was not discovered by chance. A further way to test for the role of outliers in insecticide resistance would be to compare their relative frequencies in deads and alives after bioassays.

### Candidate genes for resistance to deltamethrin and temephos

Among the SNPs significantly associated with resistance, only two were non-synonymous. Both were associated with resistance to temephos, and both involved genes encoding for enzymes. One induced a change from a threonine to a serine in an alkaline phosphatase while the other induced a change from a lysine to an asparagine in a sulfotransferase (Table S4). Alkaline phosphatases (ALP) catalyse the transfer of a phosphate group to water (hydrolysis) or alcohol (transphosphorylation) [32]. ALP-based assays have been used to detect pesticides in the environment, including organophosphates [33] and might thus be involved in their detoxification. Sulfotransferases are known to be involved in phase II detoxification processes by adding a sulphate group to various substrates to facilitate their excretion [34,35]. These two genes (and point mutations) were never described previously as being linked to temephos resistance, but they represent valuable candidates for further functional validation. All the other SNPs significantly associated with insecticide resistance were synonymous, and therefore not associated to protein variations potentially contributing to resistance, but rather in the regulation of gene expression (transcription factors, miRNA) or being in linkage disequilibrium with a functional mutation (hitch-hiking with the adaptive mutation). Because we genotyped individuals from natural populations, the length of linkage disequilibrium is likely to be low, and we restricted our search for candidates to 500 Kb upstream or downstream from each SNP (Table S4). Among candidates, we found several genes potentially involved in detoxification processes such as phosphatases, dehydrogenases and transferases. In mammals, alcohol and aldehyde dehydrogenases (ADH and ALDH) can contribute to the metabolism of pyrethroids through further processing of the metabolites generated by esterases, phenoxybenzyl alcool and phenoxybenzyl aldehyde, to phenoxibenzoic acid [36,37]. However, no classical detoxification enzymes found over-transcribed in insecticide-resistant populations from Martinique were identified [3,4]. Indeed, recent data suggest that cytochrome P450 monoxygenases play a crucial role in pyrethroid detoxification in mosquitoes [38] while carboxylesterases are known to confer resistance to organophosphates [39]. It is possible that mutations causing the over-transcription of these detoxification genes in Martinique are located too far away from our markers, or that regulators of detoxification enzymes are located far away from the gene they regulate (trans-regulators); indeed, we identified potential regulators of transcription situated close to SNPs associated with resistance (Table S4). Although this genome scan provides valuable data to identify insecticide resistance genes in mosquitoes, the main limits are the partial annotation of the *Ae. aegypti* genome, with many genes annotated as ‘hypothetical proteins’ of unknown function, and the poor assembly of the genome with 4,758 supercontigs. As a result, we restricted our search to genes located 500 Kb in either direction in the same supercontig as the SNP associated to resistance, a relatively short distance, that was even reduced for markers located closer than 500 Kb to supercontig boundaries (see Table S4). 

Finally, there was no SNP or genomic region associated with both resistance to deltamethrin and to temephos, which suggests that different resistance mechanisms are evolving in natural populations against these two commonly used insecticides, temephos being used against larvae and deltamethrin against adults. These two insecticides have different targets, the acetylcholinesterase and the sodium channel respectively, and the finding of different genes involved in the two types of resistance is not surprising. Functional validation of the precise role in each type of insecticide resistance for each candidate gene detected in this study is the next step to be undertaken.

## Supporting Information

Table S1 **GPS coordinates and characteristics of the 16 populations sampled in Martinique.**
(DOCX)

Table S2 **Genetic variability and goodness of fit to Hardy-Weinberg expectation in *Ae. aegypti* populations at the micro-geographic level in Martinique.**
(DOC)

Table S3 **Comparison of pairwise Fst obtained with microsatellites and SNPs for six populations of Martinique.**
(DOCX)

Table S4 **Genes up to 500 kb each direction from the SNP.**
(XLSX)
