# Supplementary material for: Insecticide-Driven Patterns of Genetic Variation in the Dengue Vector Aedes aegypti in Martinique Island
Source: PLoS One. 2013 Oct 18;8(10):e77857. doi: 10.1371/journal.pone.0077857 (PMC3799629; doi:10.1371/journal.pone.0077857)
Supplement: Table S1 — GPS coordinates and characteristics of the 16 populations sampled in Martinique. (DOCX) [file pone.0077857.s001.docx]

**Table S1.** GPS coordinates and characteristics of the 16 populations sampled in Martinique.

| **Name** | **Code** | **GPS Coordinates** | **Altitude** | **Area*** | **Environment** | **Inh. / km^2^** | **Deltamethrin**** | **Interventions***** |
| --- | --- | --- | --- | --- | --- | --- | --- | --- |
| Ajoupa-Bouillon | AJPB | 14°48'N 61°6'W | 227m | NA | Rural / Mountainous | 135 | 0 | 1 |
| Diamant | DIAM | 14°29'N 60°59'W | 18m | SC | Urban / Coastal | 204 | 7 | 4 |
| Fond St Denis | FSDN | 14°44'N 61°8'W | 280m | NC | Rural / Mountainous | 39 | na | 1 |
| Fort-de-France | FDF | 14°36'N 61°3'W | 7m | AC | Urban / Inland | 1973 | 1 | 13 |
| Gros Morne | GRMN | 14°41'N 60°59'W | 222m | CA | Rural / Inland | 197 | 0 | 5 |
| Ilet Anonyme | ILA | 14°37'N 60°51'W | 5m | SA | Rural / Islet | 0 | 0 | 0 |
| Ilet Long | ILO | 14°36'N 60°5'W | 5m | SA | Rural / Islet | 0 | 0 | 0 |
| Lamentin | LAM | 14°37'N 60°59'W | 10m | AC | Urban / Inland | 632 | 13 | 68 |
| Rivière Salée | RSAL | 14°30'N 60°59'W | 71m | SC | Rural / Inland | 332 | 5 | 48 |
| St Anne | SAN | 14°26'N 60°50'W | 44m | SA | Rural / Inland | 129 | 6 | 27 |
| St Esprit | SESP | 14°33'N 61°56'W | 27m | SC | Urban / Inland | 379 | 7 | 14 |
| St Joseph | SJOS | 14°41'N 61°1'W | 160m | CA | Rural / Inland | 397 | 0 | 5 |
| St Pierre | SPIER | 14°44’N 61°10'W | 9m | NC | Urban / Coastal | 117 | 12 | 8 |
| Ste Marie | SMAR | 14°47'N 60°59'W | 4m | CA | Urban / Coastal | 432 | 8 | 3 |
| Trinité | TRIN | 14°45'N 60°54'W | 177m | CA | Rural / Peninsula | 296 | 39 | 14 |
| Vauclin | VCLN | 14°34'N 60°51'W | 5m | SA | Rural / Coastal | 225 | 2 | 15 |

*CA: Center Atlantic, NA: North Atlantic, SA: South Atlantic, SC: South Caribe, AC: Agglomeration Center. **Deltamethrin space spraying treatments between 2006 and 2009. ***Interventions: source reduction including temephos treatments (but not deltamethrin treatments performed on adults) between 2006 and 2009. na: not available
