# Supplementary material for: Insecticide-Driven Patterns of Genetic Variation in the Dengue Vector Aedes aegypti in Martinique Island
Source: PLoS One. 2013 Oct 18;8(10):e77857. doi: 10.1371/journal.pone.0077857 (PMC3799629; doi:10.1371/journal.pone.0077857)
Supplement: Table S2 — Genetic variability and goodness of fit to Hardy-Weinberg expectation in Ae. aegypti populations at the micro-geographic level in Martinique. (DOC) [file pone.0077857.s002.doc]

**Table S2.** Genetic variability and goodness of fit to Hardy-Weinberg expectation in *Ae. aegypti* populations at the micro-geographic level in Martinique

|  |  | Fort de France | | |  |  |  |  |  |  |  |  | Sainte Anne | | |  | Vauclin | |  |  |  |
| --- | --- | --- | --- | --- | --- | --- | --- | --- | --- | --- | --- | --- | --- | --- | --- | --- | --- | --- | --- | --- | --- |
|  |  | 8 | 9 | 10 | 11 | 12 | 13 | 14 | 15 | 16 | 17 | all | 22 | 23 | 24 | 25 | 26 | 27 | 28 | 29 | all |
|  |  | n=30 | n=30 | n=30 | n=30 | n=26 | n=30 | n=30 | n=30 | n=30 | n=30 |  | n=30 | n=30 | n=30 | n=30 | n=30 | n=30 | n=30 | n=29 |  |
| **34/72** | *N_all_* | 2 | 4 | 4 | 2 | 2 | 2 | 3 | 2 | 3 | 3 |  | 2 | 2 | 2 | 3 | 2 | 2 | 2 | 3 |  |
|  | *R_s_* | 1.71 | 3.14 | 3.09 | 1.56 | 2.00 | 2.00 | 2.36 | 2.00 | 2.40 | 2.73 |  | 1.78 | 1.90 | 1.92 | 2.42 | 2.00 | 2.00 | 2.00 | 2.10 |  |
|  | *F_IS_* | 0.659 | **0.633** | **0.810** | -0.018 | 0.351 | -0.364 | -0.352 | -0.415 | 0.291 | **0.608** | **0.176** | -0.042 | 0.357 | -0.094 | -0.206 | -0.515 | -0.600 | -0.515 | -0.043 | -0.490 |
| **Aedga** | *N_all_* | 1 | 1 | 1 | 1 | 1 | 1 | 1 | 1 | 1 | 3 |  | 1 | 1 | 1 | 3 | 1 | 1 | 1 | 1 |  |
|  | *R_s_* | 1.00 | 1.00 | 1.00 | 1.00 | 1.00 | 1.00 | 1.00 | 1.00 | 1.00 | 1.77 |  | 1.00 | 1.00 | 1.00 | 1.67 | 1.00 | 1.00 | 1.00 | 1.00 |  |
|  | *F_IS_* | NA | NA | NA | NA | NA | NA | NA | NA | NA | 0.500 | **0.500** | NA | NA | NA | **0.500** | NA | NA | NA | NA | - |
| **A10** | *N_all_* | 4 | 4 | 4 | 3 | 3 | 4 | 3 | 4 | 4 | 4 |  | 3 | 4 | 3 | 3 | 3 | 4 | 3 | 5 |  |
|  | *R_s_* | 3.42 | 3.21 | 3.27 | 2.71 | 2.99 | 2.67 | 2.33 | 3.78 | 3.23 | 3.75 |  | 2.71 | 3.81 | 2.56 | 2.81 | 2.81 | 3.10 | 2.33 | 4.26 |  |
|  | *F_IS_* | 0.264 | 0.163 | -0.163 | -0.371 | -0.416 | 0.077 | -0.023 | 0.003 | -0.103 | 0.040 | -0.022 | -0.037 | 0.029 | 0.201 | -0.299 | 0.101 | -0.188 | -0.042 | 0.051 | 0.012 |
| **Aed19** | *N_all_* | 2 | 2 | 2 | 2 | 2 | 2 | 2 | 2 | 2 | 2 |  | 2 | 2 | 2 | 2 | 2 | 2 | 2 | 2 |  |
|  | *R_s_* | 2.00 | 2.00 | 2.00 | 2.00 | 2.00 | 2.00 | 2.00 | 1.85 | 2.00 | 1.99 |  | 2.00 | 2.00 | 1.97 | 2.00 | 2.00 | 2.00 | 2.00 | 2.00 |  |
|  | *F_IS_* | -0.017 | 0.321 | 0.059 | 0.000 | -0.282 | -0.244 | -0.158 | -0.061 | -0.234 | 0.051 | -0.008 | 0.198 | -0.325 | 0.151 | 0.067 | 0.231 | -0.041 | -0.182 | -0.080 | -0.016 |
| **Aedc** | *N_all_* | 2 | 2 | 2 | 2 | 1 | 2 | 2 | 2 | 3 | 2 |  | 2 | 2 | 2 | 2 | 4 | 4 | 3 | 3 |  |
|  | *R_s_* | 1.93 | 1.92 | 1.89 | 1.97 | 1.00 | 1.38 | 2.00 | 1.63 | 2.15 | 1.33 |  | 1.76 | 2.00 | 2.00 | 2.00 | 3.06 | 2.96 | 2.63 | 2.34 |  |
|  | *F_IS_* | -0.098 | -0.094 | -0.077 | 0.147 | NA | 0.000 | -0.389 | -0.020 | -0.058 | 0.000 | 0.072 | -0.040 | -0.101 | 0.164 | 0.183 | -0.008 | -0.108 | -0.115 | 0.063 | 0.040 |
| **H08** | *N_all_* | 3 | 3 | 4 | 3 | 3 | 3 | 2 | 3 | 3 | 3 |  | 3 | 3 | 3 | 3 | 2 | 3 | 2 | 3 |  |
|  | *R_s_* | 2.89 | 2.89 | 3.30 | 2.82 | 2.77 | 2.63 | 1.78 | 2.84 | 2.94 | 2.61 |  | 2.82 | 3.00 | 2.84 | 2.94 | 2.00 | 2.38 | 2.00 | 2.36 |  |
|  | *F_IS_* | -0.024 | 0.151 | **0.382** | 0.133 | 0.005 | -0.192 | -0.042 | -0.149 | -0.290 | 0.038 | **-0.155** | 0.217 | -0.199 | 0.583 | -0.177 | 0.386 | -0.378 | -0.276 | -0.271 | -0.155 |
| **Mean across all loci** | *N_all_* | 2.33 | 2.67 | 2.83 | 2.17 | 2.00 | 2.33 | 2.17 | 2.33 | 2.67 | 2.83 |  | 2.17 | 2.33 | 2.17 | 2.67 | 2.33 | 2.67 | 2.17 | 2.83 |  |
|  | *R_s_* | 2.16 | 2.36 | 2.43 | 2.01 | 1.96 | 1.95 | 1.91 | 2.18 | 2.29 | 2.36 |  | 2.01 | 2.29 | 2.05 | 2.31 | 2.15 | 2.24 | 1.99 | 2.34 |  |
|  | *F_IS_* | 0.103 | **0.270** | **0.187** | -0.066 | -0.110 | -0.169 | -0.213 | -0.143 | -0.139 | **0.167** | **0.040** | 0.087 | -0.103 | 0.271 | -0.092 | 0.029 | **-0.277** | **-0.252** | -0.057 | -0.139 |

8 : Fort-de-France 1, 9: Fort-de-France 2; 10: Fort-de-France 3; 11: Fort-de-France 4; 12: Fort-de-France 5; 13: Fort-de-France 6; 14: Fort-de-France 7; 15: Fort-de-France 8; 16: Fort-de-France 9; 17: Fort-de-France 10; 22: Sainte-Anne 1; 23: Sainte-Anne 2; 24: Sainte-Anne 3; 25: Sainte-Anne 4; 26: Vauclin 1; 27: Vauclin 2; 28: Vauclin 3; 29: Vauclin 4. *N_all_*: number of scored alleles; *R_s_*: Allele richness; *F_IS_*: Inbreeding coefficient. In bold: significant deficit in heterozygotes (P < 0.05) after Bonferroni correction.
