# Supplementary material for: Insecticide-Driven Patterns of Genetic Variation in the Dengue Vector Aedes aegypti in Martinique Island
Source: PLoS One. 2013 Oct 18;8(10):e77857. doi: 10.1371/journal.pone.0077857 (PMC3799629; doi:10.1371/journal.pone.0077857)
Supplement: Table S3 — Comparison of pairwise Fst obtained with microsatellites and SNPs for six populations of Martinique. (DOCX) [file pone.0077857.s003.docx]

**Table S3: Comparison of pairwise Fst obtained with microsatellites and SNPs for six populations of Martinique**

|  |  | LAM | RSAL | SAN | ILA | ILO |
| --- | --- | --- | --- | --- | --- | --- |
| GRMN | microsat. | **0.046** | 0.029 | 0.028 | **0.041** | 0.008 |
|  | SNP | 0.006 | 0.009 | **0.034** | **0.033** | **0.041** |
| LAM | microsat. |  | **0.085** | **0.051** | **0.116** | **0.052** |
|  | SNP |  | 0.010 | **0.033** | **0.034** | **0.037** |
| RSAL | microsat. |  |  | **0.048** | **0.099** | **0.098** |
|  | SNP |  |  | **0.034** | **0.029** | **0.043** |
| SAN | microsat. |  |  |  | **0.143** | **0.087** |
|  | SNP |  |  |  | **0.048** | **0.059** |
| ILA | microsat. |  |  |  |  | 0.035 |
|  | SNP |  |  |  |  | 0.014 |

Pairwise Fst for 6 microsatellites and 200 SNP (GENETIX software). In bold, significant *P*-values < 0.0033 (Bonferroni correction) after 300 permutations.
